# Review on Scrub Typhus: An Important Etiology of Acute Undifferentiated Fever Illness

**DOI:** 10.7759/cureus.47290

**Published:** 2023-10-18

**Authors:** Mihika Khairkar, Sunita Vagha, Vijayshri Deotale

**Affiliations:** 1 Microbiology, Jawaharlal Nehru Medical College, Datta Meghe Institute of Medical Sciences, Wardha, IND; 2 Pathology, Jawaharlal Nehru Medical College, Datta Meghe Institute of Medical Sciences, Wardha, IND; 3 Microbiology, Mahatma Gandhi Institute of Medical Sciences, Wardha, IND

**Keywords:** pyrexia of unknown origin (puo), acute undifferentiated febrile illness, vector-borne, febrile illness, scrub typhus

## Abstract

One of India's predominant public health issues is acute undifferentiated fever illness (AUFI), a typical response to an infectious agent. Diagnosis becomes challenging when the disease has been reported with fever as the primary symptom. Among the cases presenting at a tertiary care hospital in central India, 88% had an acute undifferentiated fever, the most common being dengue infection. In India, rural communities are at more risk from AUFI than cities. Most of those in danger are those who reside in remote areas, and one of the most significant risks is for those who reside close to forests. AUFI is a complex condition for physicians to deal with and is one of the most frequent clinical conditions for which empirical treatment is required. Nowadays, AUFI can be managed by a syndromic approach with the judicial use of antibiotics.

Symptoms of AUFI, along with myalgia, headache, and anorexia, can be caused by various illnesses. Patients are recommended to undertake a battery of investigations, which may delay the therapy and increase expenses because many diseases may present with the same symptoms. In the developed world, viral illness is the primary cause of AUFI. However, in developing countries like India, it can also be brought on by potentially curable but life-threatening conditions such as malaria, leptospirosis, hantavirus infection, and Japanese encephalitis. Lack of knowledge of the locally prevalent illnesses, which might be the cause of AUFI, and lack of preliminary screening and diagnostics at the point of care to identify the etiologies make it difficult to control these generally curable causes of the burden of AUFI, especially in tropical and subtropical countries. A deeper understanding of AUFI is required to develop better diagnostics and cures for various etiologies, especially scrub typhus.

## Introduction and background

Febrile illnesses are one of the leading causes of hospital visits and a typical sign of any infectious disease. Most febrile illnesses with unknown etiologies are treated generically, with antipyretics and empiric antibiotics, which compromises clinical diagnosis because the lack of diagnostic facilities in tropical areas is very limited [1]. Delayed hospital visits for the diagnosis or lack of correct diagnosis, many unpreventable deaths in patients suffering from AUFI were reported. So, it is important that depending on the cause of acute febrile illness, AUFI can be treated by general practitioners using criteria for investigation and antibiotic therapy based on the prevalent organisms and their susceptibility patterns [2]. Acute febrile illness and chronic febrile illness are terms used to describe fever illnesses based on the duration of the condition. Acute febrile illness, often known as acute fever or brief febrile illness, is any condition that is accompanied by a fever that lasts 15 days or less, manifests suddenly, and may be due to a variety of pathogens [3]. In general, infectious diseases are the leading causes of acute febrile illness.

According to the epidemiological data currently available, viral infections are first believed to be the primary cause of most acute febrile diseases. Some of these infections can be prevented with vaccination and regulated with vector control methods [4]. AUFI caused by viruses does not require antibiotic treatment unless there is a secondary bacterial infection. Various research studies have identified additional causative agents that can be treated effectively with antibiotics, such as rickettsial and leptospirosis infections [5]. For a few decades, widespread efforts have been made worldwide to control malaria with encouraging results. Bacteria is one of the primary causes of AUFI, which can be swiftly treated with antibiotic medication and pose a significant risk to each patient if left untreated, which is why testing algorithms and treatment guidelines were developed for AUFI [6,7]. In most developing nations, fever is the primary indicator of malaria and non-malarial illnesses such as dengue, leptospirosis, enteric fever, and Japanese encephalitis [8,9].

## Review

Methodology

The primary data source for this review was the PubMed database, which is a widely recognized and authoritative repository of biomedical literature. The search string used to identify relevant articles was as follows: ("scrub typhus"[MeSH Terms]) AND ("fever of unknown origin"[MeSH Terms]) AND (India). This search string was chosen to target articles specifically related to scrub typhus and its association with fever of unknown origin in the context of India. After executing the search, a total of 13 articles were retrieved from the PubMed database. These articles were thoroughly screened to ensure their relevance and suitability for inclusion in the review. 

Based on our search, it is understood that the etiology of acute febrile illness needs a thorough investigation, and a review is attempted to unravel the same. The endemic cause of acute febrile illness, which is more frequently observed, is mosquito-borne, primarily in tropical areas, and has significant morbidity, mortality, and economic costs. Patients with acute, unexplained fever in tropical areas exhibit a variety of etiologies, including leptospirosis, enteric fever, scrub typhus, rickettsioses, dengue fever, and malaria. In non-tropical regions, however, the etiology of acute undifferentiated fever is not well recognized.

The Indian situation

Acute undifferentiated fever (AUF) is a common illness that causes hospitalization of patients, especially between June and September, during the monsoon and post-monsoon seasons. The leading cause of AUFI in India is dengue, followed by malaria, enteric fever, and scrub typhus. Practically, all the parts of India are prevalent for AUFI. Studies were conducted in India to identify the etiological agents and clinical spectrum of acute undifferentiated febrile fever.

Comparison of AUFI and Pyrexia of Undetermined Origin (PUO)

It is critical to understand how an AUFI differs from pyrexia of undetermined origin (PUO), which was initially described as an illness lasting longer than three weeks with multiple episodes of a fever over 38.3 °C and no diagnosis after one week of hospital observation [10]. Further research following Griffin [9] adopts various definitions without specific signs of inflammation or any obvious local signs of febrile illness.

Etiological agents

In many regions of the world, the etiology of acute febrile diseases is still poorly understood. It is difficult to detect and treat because of the scarcity of resources in tropical areas and the number of unknown etiological agents of AUFI. These problems impede public health efforts to combat endemic and pandemic diseases [11]. Though it is frequently seen in clinical practice, the cause of acute undifferentiated fever (AUF) is not always known. Patients in tropical regions who presented with acute undifferentiated fever have been reported to have a range of etiologies. High economic loss, as well as morbidity and mortality, are brought on by acute undifferentiated fever. Acute undifferentiated fever's etiology has not been well investigated [12].

Acute febrile fever epidemics have been a significant source of worry in India. Most non-malarial illnesses, including dengue, leptospirosis, enteric fever, and Japanese encephalitis, manifest as acute, non-differentiated fevers in developing nations. These illnesses are serious public health issues. As a result, in these parts of the world, potentially serious diseases such as malaria, Japanese encephalitis, leptospirosis, rickettsiosis, enteric fever, dengue fever, and other infectious diseases are included in the differential diagnosis for AUFI [13]. However, most people with AUFI experience generalized symptoms (such as low-grade fever, malaise, headache, and muscle aches) and frequently lack a focal site of infection. Although physical symptoms lack the specificity and accuracy needed to rule in or rule out a particular infectious disease as the cause of an AUFI, history and physical examination, the traditional techniques used by health professionals worldwide, can offer crucial insights into the etiology of AUFI. A history or a physical examination is not trustworthy enough to identify the causes of acute undifferentiated fevers (Table 1).

**Table 1:** Clinical features of etiological agents

Disease	Fever	Clinical features
Dengue	Break bone fever, biphasic fever with maculopapular rash in dengue shock syndrome fever with shock	Fever, Headache, Retrobulbar pain, panini in the back and limb, maculopapular rash, shock
Malaria	Intermittent, with the spike of fever quatrain, quotidian, tertian	Fever with chills and rigors, jaundice, hepatomegaly
Enteric fever	Gradual onset, step ladder type	Fever, headache, abdominal discomfort, constipation, bradycardia, splenomegaly, rose spots
Japanese encephalitis	Acute onset high-grade fever	Fever, headache, myalgia, altered mental status, somnolence, and irritability
Scrub typhus	Acute onset fever	Fever, headache, myalgia, and eschar formation at the site of inoculation. Multiple organ dysfunction

Most individuals with AUFI have non-specific symptoms; for instance, a third of dengue patients exhibit signs of upper respiratory infections such as sore throat, stuffy nose, and cough. Malaria, dengue, enteric fever, and leptospirosis have all been associated with enlarged liver and spleen. Like these symptoms, headache, stiff neck, and other meningeal inflammatory signals are conventionally linked to meningitis; however, they are unreliable indicators of the condition [14].

Diagnostic Requirements

Due to the lack of specificity in the early laboratory results and the non-specific clinical signs of febrile illness, the cause of fever is not often immediately apparent. Then, these illnesses are referred to as undifferentiated fever, and a comprehensive differential diagnosis is available, typically affected by the patient's geographical location. Additional laboratory tests are typically conducted to ascertain the reason for the fever. Occasionally, undifferentiated fevers can go misdiagnosed despite investigation, and while some undiagnosed, undifferentiated fevers remit on their own, others may be associated with significant morbidity and fatality. The diagnosis of AUFI is frequently made using serological diagnosis, serving as the cornerstone for the final diagnosis of these disorders because of the atypical clinical symptoms and the lack of localizing indications. Missed or incorrect diagnoses of these AUFI can increase the risk of consequences [15].

Diagnostic Tests

**Figure 1:**
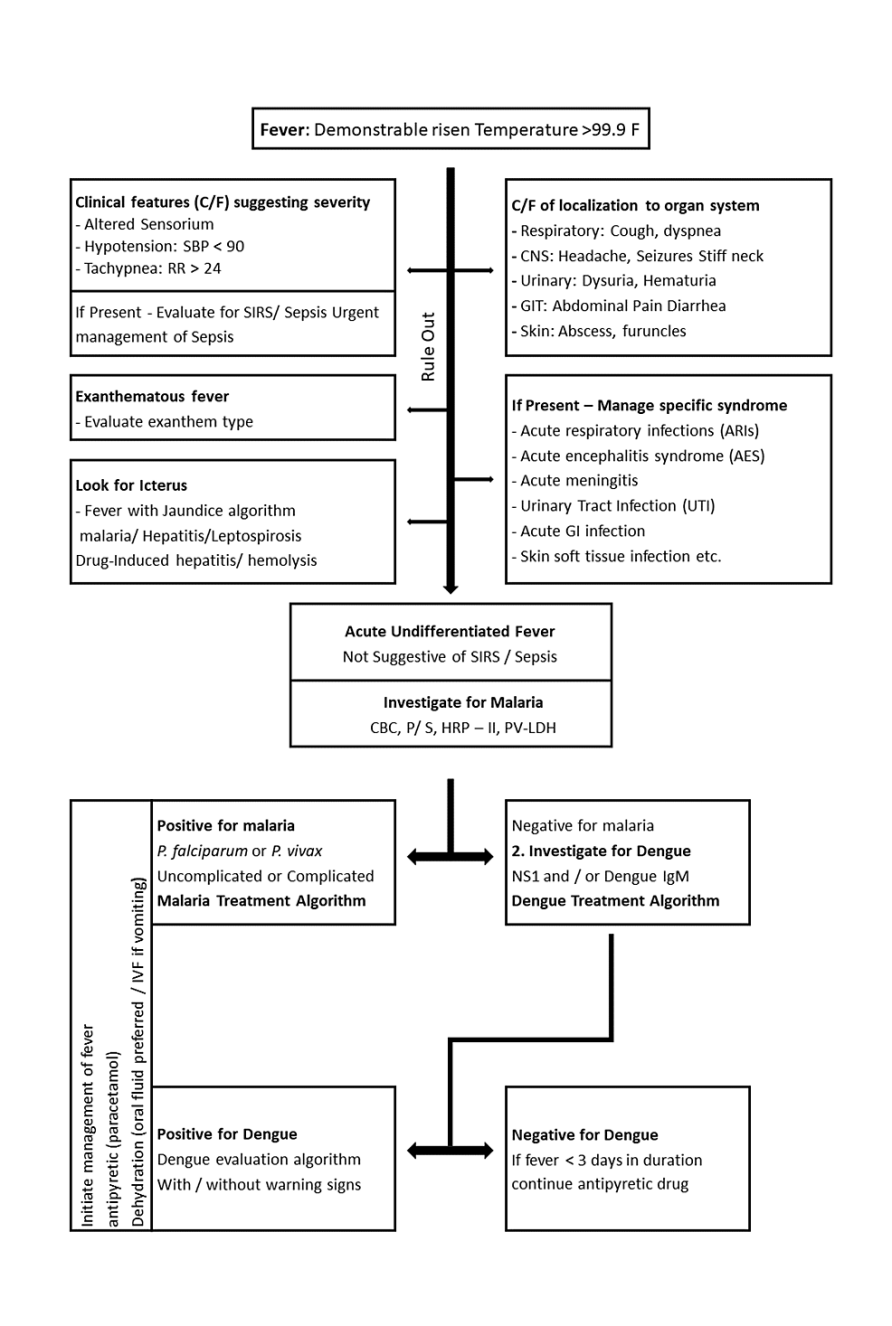
Diagnostic algorithm for acute undifferentiated febrile illnesses (AUFIs) C/F - Clinical features; SBP - systolic blood pressure; RR - respiratory rate; SIRS - systemic inflammatory response syndrome; CBC - complete blood count; PS - peripheral smear; HRP2 - histidine rich protein 2; Pv-LDH - plasmodium vivax - lactate dehydrogenase; IVF - intravenous fluid; CNS - central nervous system; GIT - gastrointestinal tract; GI - gastrointestinal; NS1 - non-structural protein; IgM - immunoglobulin M

Both non-specific and specific investigations were used in the studies to determine the etiologies of AUF. Non-specific examinations, such as a complete blood count, serum biochemistry, urinalysis, and chest X-ray, describe the underlying cause of the sickness without identifying a specific microbe. They involve blood analysis and other laboratory tests, including a specific inquiry that looks for a particular pathogen. Examples include malaria films, serological tests, polymerase chain reaction (PCR) assays, and bacterial cultures. These approaches identified specific infections, including malaria, dengue fever, leptospirosis, enteric fever, and rickettsioses, as potential causes of AUFI (Figure 1).

An unusual cause of AUFI: scrub typhus

One of the neglected causes of severe febrile illness is scrub typhus, which is caused by *Orientia tsutsugamushi* *(O. tsutsugamushi)*[16], a disease spread to people by the bite of trombiculid larva mites. *Orientia tsutsugamushi* is an obligate intracellular, gram-negative bacterium. An organism whose polysaccharides have a proteus-specific antigenic association (OXK), which is thus utilized to confirm serological assays. Typhus-infected scrub chiggers are prevalent in locations with dense scrub vegetation throughout the summer. The rainy season (therefore, the illness is sometimes known as river/flood fever) time of mite egg-laying [17] is often from June to November [18]. The vegetation that was present led to the usage of the term "scrub", which supports the chigger-mammal interaction despite the presence of rodents and mites in other areas.

An area of the world has endemic cases of scrub typhus. The "tsutsugamushi triangle" is thus named, which includes Taiwan, Japan, Nepal, Northern Pakistan, Papua New Guinea, South Korea, China, and Queensland and Northern New South Wales which are states in Australia [19,20]. Scrub typhus is a disease that has been known to exist in India for numerous years. In Assam and West Bengal, the illness affected soldiers during World War II and the Indo-Pak War of 1965. There was an increase in illness in 1990 in an army regiment stationed at the Pakistan border with India. Scrub typhus is prevalent in the monsoon or after the monsoon. Several differential diagnoses are considered to present when a patient exhibits acute febrile sickness. One significant cause is included in the differential diagnosis: scrub typhus. Even if the infection is quite subtle in the early years, a common cause of acute febrile illness today is scrub typhus [21]. The disease either presents with or without eschar. Many patients are overlooked because clinical presentations differ from the actual presentation due to the presence of eschar. After all, in some circumstances, the vector bite may not be noticeable or obvious. Sometimes, eschar might be mistaken for an insect bite or an abrasion.

If untreated, patients begin to display signs of a systemic infection the second week after the onset [22,23]. Acute diffuse encephalomyelitis, encephalopathy, meningitis, deafness, cranial nerve palsies, eye manifestations, rhythm abnormalities, myocardial involvement with congestive heart failure, vasculitis, acute renal failure, interstitial pneumonia, acute respiratory distress syndrome, and gastrointestinal system are just a few of the organ systems that can be affected by this stage of the illness (alterations in liver functions, pancreatitis, diarrhea). If not promptly treated, multi-organ dysfunction syndrome (MODS) can occasionally be fatal. Using clinical signs alone, scrub typhus is challenging to diagnose. *O. tsutsugamushi* has numerous serotypes, and infections with any one of them temporarily provide cross-immunity against another. It is a zoonotic illness spread by mites of the *Leptotrombidium deliense* group in their larval stage (chiggers). The wild rats in the subgenus Rattus are the primary food source for these larval mites. When a man enters the mite islands, which are mite-infested places, he unintentionally contracts an infection [24]. One million cases of scrub typhus occur each year, making it one of the most prevalent life-threatening rickettsial illnesses [25]. The information from this review's insights will help policymakers make logical decisions and initiate efforts to successfully control and stop the spread of this untreated disease [26,27]. It can also help in establishing a surveillance system to monitor this disease. In this review, we discussed that scrub typhus is widespread in India and typically damages many organs in individuals who test positive, raising the likelihood of complications and death. Future efforts to lessen this hazard to public health can include raising knowledge of the disease, expanding testing options, and starting the proper treatment at the right time.

Scrub Typhus Laboratory Diagnosis

For laboratory diagnosis of scrub typhus, the preferred specimens are skin, lymph node biopsy, heparinized blood, ethylenediaminetetraacetic acid (EDTA) blood, and serum. Many tests are available for laboratory diagnosis of scrub typhus: Weil-Felix test [28], indirect immunofluorescence assays (IFA) [29], indirect immunoperoxidase assays [30], enzyme-linked immunosorbent assay (ELISA), immunochromatographic tests (ICT). Among all serological assays, the IgM ELISA-based method is standard for diagnosing scrub typhus. The best method to diagnose scrub typhus is by conducting serological methods [31].

Detection of IgM /IgG Antibodies

The evidence of specific antibodies, either IgM or IgG, against scrub typhus group orientate (STGO) indicates current or past infection. Immunoassays have been developed to detect those specific antibodies using whole cells or recombinant antigens. The presence of IgM antibodies during the first week of infection indicates acute infection. The presence of group-specific IgM antibodies to the various strains of *O. tsutsugamushi* provides strong evidence of recent active infection. The sensitivity and specificity of IFA with paired sera have been reported to be 85% and 98% for IgM, taking a 1:400 cut-off, while the presence of IgG antibodies infers the past or remote infection. The presence of IgG antibodies also informs the scrub typhus in the population.

Cell Culture

In vitro culturing of *Orientia tsutsugamushi* requires a biosafety level three facility to propagate the bacterial cells using the yolk sac of embryonated chicken eggs and established cell lines in a standard fibroblast cell line of the mouse, HeLa, BHK21, Vero lineage isolated from kidney epithelial cells of African green monkey. The advantage of doing a cell culture is that it improves the sensitivity of the diagnosis method by propagating cells to increase sample density. Bio-safety level 3 is required for the cell culture.

Immunochromatography Test (ICT)

ICT is a rapid diagnostic test for diagnosing scrub typhus. Due to the ability to produce high-quality protein, antigens have become more specific, which helps in the superior positive predictive value of the diagnosis. The advantage of doing this test is it is a Point of Care diagnostic test and does not require a sophisticated instrument. It can be performed in various limited healthcare resource settings. This test has less sensitivity and specificity due to higher antigenic diversity amongst the serotypes [31].

Immunofluorescence Assay (IFA)

IFA has higher sensitivity, specificity, and higher accuracy, which is why it is considered the gold-standard test for scrub typhus. The IFA test uses fluorescent-linked anti-human reporter antibodies to detect the presence of scrub typhus-specific antibodies in the serum sample. Karp, Kato, and Gilliam's are the commonly used antigens in indirect IFA for the immunofluorescence test. To label a test as positive, no accurate antibody cut-off is there [31].

Weil-Felix Test

This heterophile antibody test is based on the antigens shared by Rickettsia and Proteus [32]. It exhibits agglutinins to *Proteus mirabilis* strain OXK and the *Proteus vulgaris* strains OX19, OX2, and OXK. Despite its low sensitivity and specificity, this test is a helpful and affordable diagnostic tool for identifying rickettsial disease in the laboratory. Only five to seven days following the commencement of the fever should this test be performed. A titer of 1:80 is regarded as indicative of infection. However, it is necessary to standardize baseline titers for every region [31].

Polymerase Chain Reaction (PCR)

PCR is currently the quickest diagnostic procedure. It can discover rickettsial DNA in whole blood, buffy coat fractions, and tissue samples [33]. The real-time format PCR focuses on the crucial 56 kDa and 47 kDa surface antigen genes [31].

Treatment and Prevention

The preferred medications for treating scrub typhus are tetracycline (doxycycline) and chloramphenicol, which work equally well when administered orally or intravenously [34,35]. Another great option is azithromycin, especially if doxycycline resistance is thought to exist. Although rifampicin is used as a second-line treatment, it should only be administered after active tuberculosis has been ruled out due to the danger of developing drug-resistant tuberculosis. There is not a vaccine for scrub typhus yet. Therefore, prevention centers mostly on preventing insect bites.

## Conclusions

In the current era of antibiotic resistance, the defined approach to handle AUFI is necessary because empirical treatment frequently results in developing resistance. When AUFI is adequately handled, investigations and the use of antibiotics are both made more effective. Antibiotic resistance and cost are decreased as a result. The majority of the AUFI may be accurately predicted by recording the correct history and a thorough physical examination and laboratory tests, as was demonstrated in our study. Therefore, it is beneficial for all laboratories to have the tests mentioned above available during epidemic seasons. Because antibody titers are below detectable levels in the early stages of the disease, serological testing with only acute sera frequently produces false negative results, especially in tests that try to detect antibodies against infection. Since most of the focus is on hospitalized patients, one needs to extrapolate to milder forms of AUFI that occur in the community, and we miss a substantial part of AUFI patients who were seeking outpatient department (OPD) base care and therapy, which may lead to underestimating the actual scrub typhus burden.
